# Loss of GCN5L1 in cardiac cells limits mitochondrial respiratory capacity under hyperglycemic conditions

**DOI:** 10.14814/phy2.14054

**Published:** 2019-04-29

**Authors:** Dharendra Thapa, Manling Zhang, Janet R. Manning, Danielle A. Guimarães, Michael W. Stoner, Yen‐Chun Lai, Sruti Shiva, Iain Scott

**Affiliations:** ^1^ Division of Cardiology Department of Medicine University of Pittsburgh Pittsburgh Pennsylvania; ^2^ Department of Pharmacology and Chemical Biology University of Pittsburgh Pittsburgh Pennsylvania; ^3^ Vascular Medicine Institute Department of Medicine University of Pittsburgh Pittsburgh Pennsylvania; ^4^ Center for Metabolism and Mitochondrial Medicine Department of Medicine University of Pittsburgh Pittsburgh Pennsylvania; ^5^ Division of Pulmonary Critical Care, Sleep and Occupational Medicine Department of Medicine Indiana University School of Medicine Indianapolis Indiana

**Keywords:** bioenergetics, GCN5L1, hyperglycemia, mitochondria, respiration, SIRT3

## Abstract

The mitochondrial acetyltransferase‐related protein GCN5L1 controls the activity of fuel substrate metabolism enzymes in several tissues. While previous studies have demonstrated that GCN5L1 regulates fatty acid oxidation in the prediabetic heart, our understanding of its role in overt diabetes is not fully developed. In this study, we examined how hyperglycemic conditions regulate GCN5L1 expression in cardiac tissues, and modeled the subsequent effect in cardiac cells in vitro. We show that GCN5L1 abundance is significantly reduced under diabetic conditions in vivo, which correlated with reduced acetylation of known GCN5L1 fuel metabolism substrate enzymes. Treatment of cardiac cells with high glucose reduced *Gcn5l1* expression in vitro, while expression of the counteracting deacetylase enzyme, *Sirt3*, was unchanged. Finally, we show that genetic depletion of GCN5L1 in H9c2 cells leads to reduced mitochondrial oxidative capacity under high glucose conditions. These data suggest that GCN5L1 expression is highly responsive to changes in cellular glucose levels, and that loss of GCN5L1 activity under hyperglycemic conditions impairs cardiac energy metabolism.

## Introduction

Growing evidence suggests that lysine acetylation, a reversible post‐translational modification, regulates mitochondrial bioenergetic output. Several proteomics studies have found that numerous mitochondrial metabolic enzymes are acetylated (Kim et al. 2006; Zhao et al. 2010; Choudhary et al. 2014), and the regulation of fuel metabolism enzyme activity by acetylation appears to be particularly important (Kim et al. 2006; Zhao et al. 2010; Choudhary et al. 2014) in energy‐demanding tissues. Mitochondrial protein acetylation is significantly increased in various tissues during obesity (Hirschey et al. 2010; Alrob et al. 2014; Thapa et al. 2017), and circulating fatty acids are the main source of the acetyl‐CoA used for lysine acetylation (Pougovkina et al. 2014). These data imply that there is an intrinsic link between nutritional inputs, lysine acetylation, and metabolic activity, and suggests that dysregulation of this post‐translational modification could be a key mechanism in the development of metabolic dysfunction.

The regulation of mitochondrial lysine acetylation has predominantly focused on the activity of SIRT3, the major mitochondrial deacetylase enzyme (Schwer et al. 2002). SIRT3 has been shown to target a number of different regulators of energy metabolism, including the fatty acid oxidation enzymes long‐chain acyl‐CoA dehydrogenase (LCAD) and hydroxyacyl‐CoA dehydrogenase/3‐ketoacyl‐CoA thiolase/enoyl‐CoA hydratase alpha subunit (HADHA) (Alrob et al. 2014). GCN5L1 is localized in the mitochondrial matrix, and has been shown to counter the activity of SIRT3 and acetylate these two enzymes (Scott et al. 2012; Thapa et al. 2017, 2018). Deletion of GCN5L1 reduces mitochondrial protein acetylation and bioenergetic output, which promotes mitochondrial dysfunction and cellular energy depletion in murine fibroblasts (Scott et al. 2014).

While our knowledge of mitochondrial protein acetylation and the control of its regulatory enzymes in obesity and prediabetes are rapidly expanding, our understanding of their function during overt diabetes remains limited. In this study, we examined how GCN5L1 and SIRT3 are regulated in the hearts of diabetic ZSF1 rats, and then modeled these changes in vitro. Under hyperglycemic conditions we found that the abundance of SIRT3 remained unchanged, while GCN5L1 was significantly downregulated. This correlated with reduced acetylation of known GCN5L1 fuel metabolism enzymes. Genetic depletion of GCN5L1 in H9c2 cells resulted in decreased glucose‐driven mitochondrial respiration, which became pronounced during hyperglycemia. We conclude that GCN5L1 is highly responsive to changes in cardiac glucose levels, and that loss of GCN5L1 expression may be a causative factor in the development of metabolic dysfunction under hyperglycemic conditions.

## Methods

### Animal care and use

Male lean and obese ZSF1 rats were housed in the University of Pittsburgh animal facility. Experiments were conducted in compliance with National Institutes of Health guidelines, and followed procedures approved by the University of Pittsburgh Institutional Animal Care and Use Committee. Banked frozen cardiac tissues from a previous study (Lai et al. 2016) were used to analyze the expression of mitochondrial proteins under lean (nondiabetic) and obese (diabetic conditions).

### Protein isolation

For whole cardiac protein lysate, tissues were minced and lysed in CHAPS buffer on ice for ~2 h. Homogenates were spun at 10,000***g***, and the supernatants collected for western blotting or co‐immunoprecipitation experiments.

### Western blotting and co‐immunoprecipitation

Protein lysates were prepared in LDS sample buffer, separated using Bolt SDS/PAGE 4–12% or 12% Bis‐Tris gels, and transferred to nitrocellulose membranes (Life Technologies). Protein expression was analyzed using the following primary antibodies: rabbit SIRT3, rabbit acetyl‐lysine (Ac‐K), rabbit glutamate dehydrogenase (GDH), mouse tubulin, and rabbit pyruvate dehydrogenase (PDH) from Cell Signaling Technologies; rabbit phospho‐PDH (Ser 293) from Novus; GCN5L1 as reported previously (Scott et al. 2012). Fluorescent anti‐mouse or anti‐rabbit secondary antibodies (red, 700 nm; green, 800 nm) from LiCor were used to detect expression levels. For co‐immunoprecipitation experiments, protein lysates were harvested in CHAPS buffer, and equal amounts of total protein were incubated overnight at 4°C with the relevant antibody or an IgG control. Immunocaptured proteins were isolated using Protein‐G agarose beads (Cell Signaling Technology), washed multiple times with CHAPS buffer and then eluted in LDS sample buffer at 95°C. Samples were separated on 12% Bis‐Tris Bolt gels and probed with appropriate antibodies. Protein densitometry was measured using Image J software (National Institutes of Health, Bethesda, MD). Protein loading was further confirmed using GDH or tubulin loading controls where appropriate.

### RNA isolation and qRT‐PCR

For qRT‐PCR, mRNA was isolated using a total RNA extraction kit (Qiagen), and cDNA was produced using a first‐strand synthesis kit (Invitrogen). Transcript levels were measured using validated gene‐specific primers (Qiagen). Each experiment was carried out at least three times, and representative results are shown. *Gapdh* was used as the internal control.

### GCN5L1 knockdown stable cell lines

H9c2 cells were purchased from ATCC (Manassas, VA). Cells were transduced (MOI = 2) with Mission lentiviral shRNA particles targeting GCN5L1 or a scrambled control sequence. Transduced cells were selected using 1.25 μg/mL puromycin (determined following a kill‐curve experiment), and cultured in this concentration for several passages. Stable cell lines were verified by qRT‐PCR and western blot for gene knock down efficiency.

### Mitochondrial bioenergetics measurements

Oxygen consumption rate (OCR) was measured in GCN5L1 control and knockdown stable H9c2 cell lines using the Seahorse XF system (Seahorse Bioscience). Cells (5 × 10^3^ cells/well) in each condition were plated and allowed to attach overnight in DMEM. On the assay day, media was changed to unbuffered DMEM media supplemented with either 5 mmol/L glucose (low glucose) or 25 mmol/L glucose (high glucose), 2 mmol/L glutamine and 0.5 mmol/L carnitine, and the plate incubated in a non‐CO_2_ incubator (37°C; 15 min) before running. Basal OCR in each well was measured, followed by serial treatment with FCCP (7.5 μmol/L) and rotenone (2 μmol/L). After completion, viability was assessed by crystal violet (CV) staining, and OCR normalized to cell number. Each experiment was repeated to ensure reproducibility, and the data presented are technical replicates (*N* = 8) of a single representative study.

### Statistics

Means ± SEM were calculated for all data sets. Data were analyzed using two‐tailed student's *t*‐tests or one‐way ANOVA (with Tukey post‐hoc tests) as appropriate. *P* < 0.05 was considered significant.

## Results

### GCN5L1 abundance and activity is reduced in diabetic ZSF1 rat hearts

**Figure 1:**
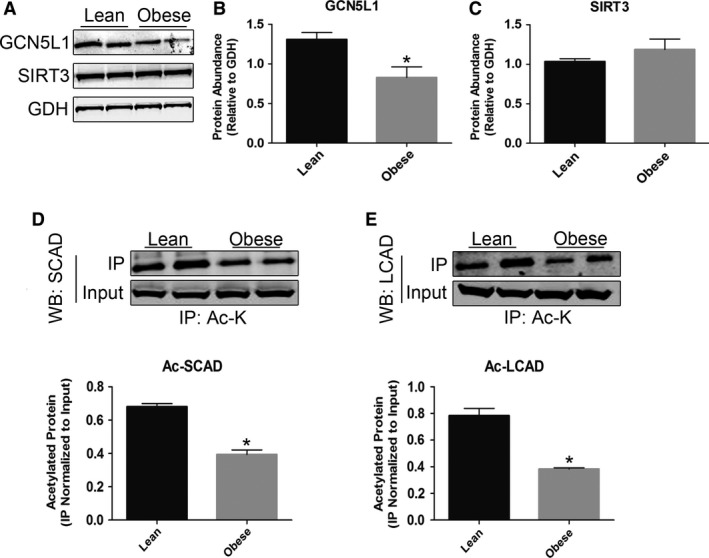
Expression of mitochondrial acetylation regulatory enzymes in ZSF1 rat hearts. (A‐C) The abundance of the mitochondrial acetyltransferase‐related protein GCN5L1 was significantly reduced in obese (diabetic) ZSF1 rats relative to lean (nondiabetic) controls. The expression of the deacetylase enzyme SIRT3 was not affected under the same conditions. (D–E) The acetylation status of two known mitochondrial GCN5L1 substrates, SCAD and LCAD, are greatly reduced in obese ZSF1 rat hearts relative to lean controls. *N* = 3, *P < 0.05.

The ZSF1 rat strain has been widely characterized as a genetic model of metabolic syndrome, with obese animals developing hyperglycemia, hyperinsulinemia, and cardiac dysfunction relative to their lean littermate controls (Lai et al. 2016). To begin investigating whether these outcomes had any effect on the regulation of mitochondrial function, we examined the expression of cardiac enzymes involved in the control of mitochondrial protein acetylation, a key regulatory system in numerous cellular metabolic pathways (Kim et al. 2006; Zhao et al. 2010; Choudhary et al. 2014). The expression of acetyltransferase‐related protein GCN5L1 in the hearts of obese rats (which display a 3‐fold increase in blood glucose levels; Lai et al. 2016) was significantly decreased relative to lean controls, while the abundance of the deacetylase enzyme SIRT3 was unchanged (Fig. 1A–C). This suggests that exposure to increased plasma nutrient levels negatively impacts GCN5L1 expression in cardiac tissue.

To investigate whether reduced expression in obese ZSF1 rats had an impact on GCN5L1 function, we examined the acetylation status of two known GCN5L1 substrates in a subset of lean and obese tissue samples. We previously showed that the acetylation status of SCAD and LCAD, two acyl‐CoA dehydrogenase enzymes in the mitochondrial fatty acid oxidation pathway, correlated with GCN5L1 expression levels (Scott et al. 2012). Consistent with this, we found that the acetylation of these enzymes was markedly reduced in obese animals displaying lower GCN5L1 levels, relative to lean controls (Fig. 1D and E). This suggests that the acetylation status of mitochondrial fuel metabolism enzymes is correlated with changes in GCN5L1 expression.

### Acute exposure to high glucose limits GCN5L1 expression in cardiac H9c2 cells

**Figure 2:**
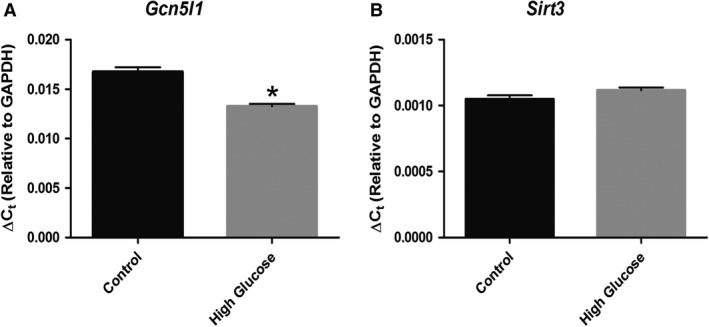
Expression of mitochondrial acetylation regulatory enzymes after acute exposure to hyperglycemia. (A‐B) Exposure of rat cardiac H9c2 cells to high glucose (25 mmol/L) for 8 h led to a significant decrease in *Gcn5l1* gene expression, while *Sirt3* expression was not affected. *N* = 4, *P < 0.05.

To examine whether these changes were related to glucose exposure or other related metabolic effects, we measured the expression of these proteins in H9c2 cells following an acute glucose treatment in vitro. In cells exposed to high glucose levels (25 mmol/L) for 8 h, there was a significant decrease in the expression of *Gcn5l1*, while *Sirt3* transcript levels remained unchanged (Fig. 2A and B). These data suggest that GCN5L1 expression is constrained in cardiac cells exposed to high levels of glucose.

### Stable knockdown of GCN5L1 does not affect mitochondrial protein expression in H9c2 cells

**Figure 3:**
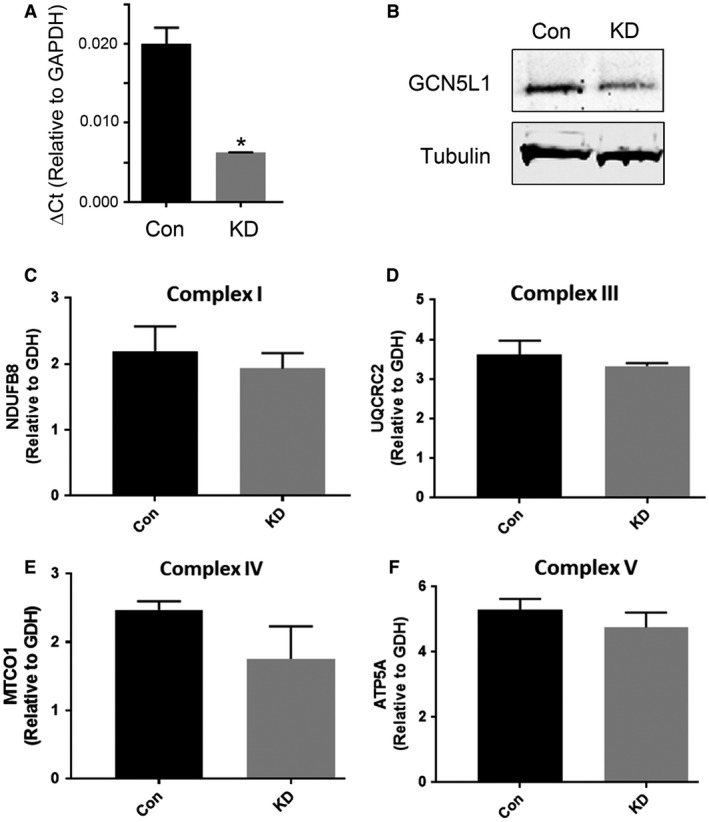
GCN5L1 knockdown does not affect expression of mitochondrial proteins. (A–B) Stable knockdown of GCN5L1 in rat H9c2 cells was confirmed by qPCR and western blot. (C–F) Western blot analysis demonstrated that there were no significant changes to the expression of several mitochondrial electron transport chain proteins in GCN5L1 knockdown cells relative to the control. *N* = 4, *P < 0.05.

Loss of GCN5L1 expression in cardiac cells has been linked to several metabolic defects, including reduced fatty acid oxidation (FAO) activity in response to decreased FAO enzyme acetylation (Alrob et al. 2014; Thapa et al. 2017). To further model the effects of GCN5L1 depletion in vitro, we generated control and GCN5L1 knockdown cell lines stably expressing nontargeting or GCN5L1 shRNA via lentiviral transduction. These cells demonstrated significant reductions in GCN5L1 expression at both the gene and protein level (Fig. 3A and B).

We previously demonstrated that deletion of GCN5L1 in mouse embryonic fibroblasts impacted mitochondrial turnover rates, and both PGC‐1α‐driven biogenesis and TFEB‐driven mitophagy are increased in GCN5L1 null cells (Scott et al. 2014). To examine if genetic depletion of GCN5L1 in H9c2 cells led to a decrease in mitochondrial proteins, we examined the expression of several electron transport chain enzymes whole cell lysates from control and knockdown cells. Despite a significant decrease in GCN5L1 expression, there was no reduction in protein markers from either complex I (NDUFB8), complex III (UQCRC2), complex IV (MTCO1), or the ATP synthase (ATP5a) (Fig. 3C‐F). In addition, there was no significant change in glutamate dehydrogenase expression in GCN5L1 knockdown cells relative to the control (data not shown), which overall suggests that there is no impact on mitochondrial volume in GCN5L1 depleted cells.

### Oxidative respiratory capacity is reduced in cardiac cells with diminished GCN5L1 expression

**Figure 4:**
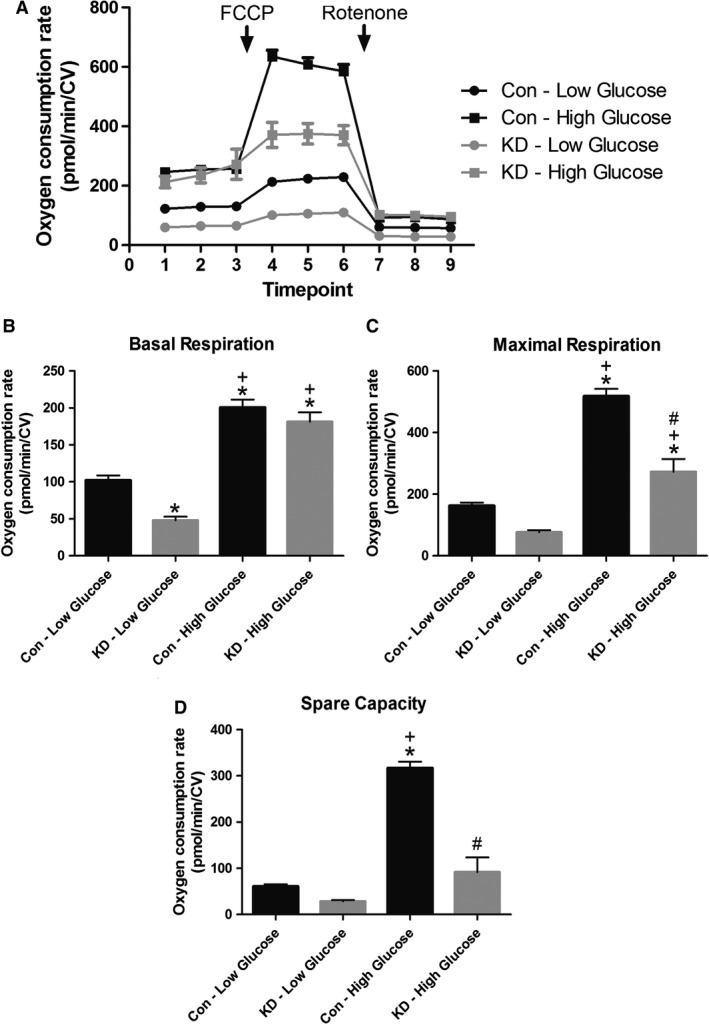
Glucose‐driven oxidative respiratory capacity in cells with diminished GCN5L1 expression. (A–D) GCN5L1 shRNA (KD) H9c2 cells displayed reduced basal respiration at low glucose levels (5 mmol/L), and significantly reduced maximal respiration and spare capacity at high glucose (25 mmol/L), compared to nontargeting control shRNA (Con) cells. *N* = 8, *P < 0.05 versus Con‐Low Glucose, +*P* < 0.05 versus KD‐Low Glucose, #*P* < 0.05 versus Con‐High Glucose.

To investigate whether there is a functional consequence of reduced GCN5L1 expression in the context of glucose‐driven respiration, we examined the mitochondrial bioenergetic profile of H9c2 cells (Fig. 4A). Under basal conditions, there was a significant decrease in the respiratory use of oxygen in GCN5L1 knockdown (KD) cells exposed to low glucose compared to control cells. However, this difference was lost when cells were exposed to high glucose levels (Fig. 4B). Conversely, under conditions that promote uncoupled respiration (treatment with the proton ionophore FCCP), there was a significant decrease observed in oxidative phosphorylation in the KD group relative to control cells under high glucose conditions only (Fig. 4C). This trend resulted in a significant decrease in respiratory spare capacity in high glucose‐treated KD cells which was not observed under low glucose conditions (Fig. 4D). These findings suggest that reduced GCN5L1 expression limits the respiratory capacity of cardiac cells, and that this is exacerbated by stress during exposure to elevated glucose levels.

### Reduced expression of GCN5L1 in H9c2 cells does not impact pyruvate dehydrogenase PTMs

**Figure 5:**
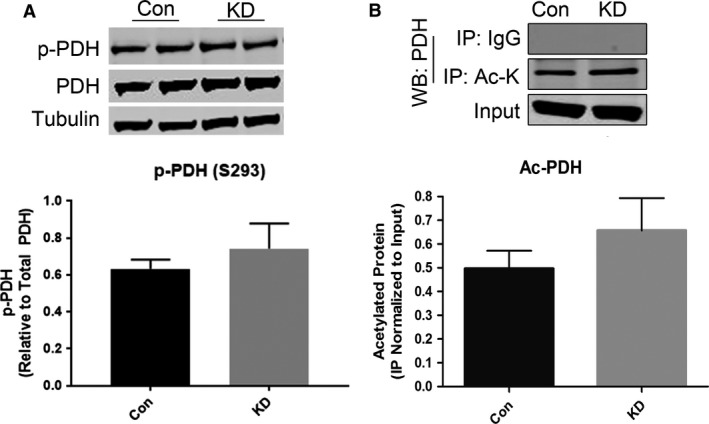
Post‐translational modifications of pyruvate dehydrogenase in GCN5L1‐depleted cells. (A–B) Under high glucose (25 mmol/L) conditions, there was no significant difference in the levels of PDH phosphorylation (S293) or acetylation between control (Con) and knockdown (KD) H9c2 cells. *N* = 4.

The mitochondrial pyruvate dehydrogenase (PDH) complex converts imported pyruvate into acetyl‐CoA for use in the TCA cycle, post‐translational modifications, and metabolite biosynthesis in numerous cell types. The activity of PDH is regulated by several post‐translational modifications (PTMs), with phosphorylation and acetylation being linked to a decrease in complex function (Mori et al. 2013). To investigate if reduced GCN5L1 expression impacts glucose oxidation via changes in PDH, we examined the phosphorylation and acetylation status of the PDH E1‐alpha subunit in control and GCN5L1 KD cells. Under conditions of elevated glucose, there was no significant difference in either the phosphorylation or acetylation status of PDH in GCN5L1 KD cells relative to the control (Fig. 5A and B). These data indicate that any changes in glucose utilization in GCN5L1 depleted cells are not likely to be related to known post‐translational modifications of PDH.

## Discussion

Mitochondrial protein acetylation is controlled by the activity of opposing acetyltransferase and deacetylase enzymes in response to changing nutritional conditions (Kim et al. 2006; Hirschey et al. 2010; Alrob et al. 2014; Thapa et al. 2017). To understand the regulatory role played by the acetyltransferase‐related protein GCN5L1 under nutrient stress, we examined mitochondrial function under hyperglycemic conditions in vivo and in vitro. In these studies, we found that: (Kim et al. 2006) acute and chronic exposure to high glucose reduces GCN5L1 expression (Zhao et al. 2010) reduced GCN5L1 expression correlates with reductions in mitochondrial metabolic enzyme acetylation; and (Choudhary et al. 2014) GCN5L1 depleted cells have reduced respiratory capacity in response to high glucose conditions. These findings suggest that GCN5L1 may play an important role in regulating mitochondrial function in the heart, and that varying nutrient conditions have an impact on GCN5L1 activity.

The transition from the prediabetic to overt diabetic state in obesity leads to significant changes in cardiac energy metabolism, which may promote contractile dysfunction and heart failure (Fillmore et al. 2014). These include an increase in fatty acid oxidation, and a decrease in the use of glucose as an oxidation substrate, which can occur through multiple mechanisms (including increased myocardial insulin resistance, the presence of elevated circulating lipids and Randle Cycle inhibition of glucose metabolism; 14). We have previously shown that in a murine model of prediabetes, increased expression of GCN5L1 in the heart promoted the acetylation and increased activity of mitochondrial fatty acid oxidation proteins (Thapa et al. 2017). In particular, increased acetylation of LCAD correlated with elevated enzyme activity in heart lysates in vitro, which was lost when GCN5L1 was knocked‐down in H9c2 cells (Thapa et al. 2017). In contrast, in the liver and other tissues, acetylation of LCAD leads to decreased fatty acid oxidation (Bharathi et al. 2013), and this discrepancy has yet to be adequately resolved. Further work is required to understand if this difference is the result of the different physiology of these two organs, or whether the lysine sites modified in the heart are different to those found in the liver (Lys‐381/Lys‐322; Bharathi et al. 2013). In this study we demonstrate that GCN5L1 abundance correlates with cardiac fatty acid enzyme acetylation. However, in the ZSF1 rat model used currently, both are reduced under overt diabetic conditions (Fig. 1), which tallies with a reduction in *Gcn5l1* gene expression in H9c2 cells incubated under hyperglycemic conditions (Fig. 2). As such, in this model, downregulation of GCN5L1 may mimic the hepatic state and promote increased fatty acid oxidation activity, and thereby aiding the switch to enhanced cardiac fatty acid utilization seen in diabetes (Fillmore et al. 2014). As an alternative, the changes in GCN5L1 expression observed may reflect a continuum whereby under conditions of prediabetes and excess nutrition, GCN5L1 becomes elevated in the heart, and that this promotes a semi‐adaptive ability to continue mitochondrial fuel substrate oxidation (in this case, fatty acids) when glucose oxidation is diminished. Once aberrant fuel metabolism reaches a certain level and overt diabetes develops, as in the ZSF1 obese rat, GCN5L1 expression is down‐regulated leading to an overall decrease in mitochondrial fuel oxidation, which particularly affects the oxidation of glucose (Fig. 4). As such, these data would suggest that the downregulation of GCN5L1 under nutrient excess may be a mechanism by which any fuel metabolism‐driven contractile dysfunction could develop in the heart.

While significant mitochondrial respiratory dysfunction develops in GCN5L1 knockdown cells only under hyperglycemic conditions, there is also a noticeable decrease in glucose‐driven mitochondrial respiration under normoglycemia (Fig. 4). This would suggest that loss of GCN5L1 either impacts glucose import, or that reduced GCN5L1 levels lead to a change in glucose handling that results in nonmitochondrial glucose utilization. The end‐product of glycolysis, pyruvate, is imported into the mitochondria via two mitochondrial pyruvate carrier (MPC) proteins, MPC1 and MPC2 (Hildyard and Halestrap 2003; Bricker et al. 2012). Previous studies in several cell types have shown that both MPCs are regulated by lysine acetylation. In colon cancer cells, hyperacetylation leads to a decrease in MPC1 activity, which is reversed by the deacetylase SIRT3 (Liang et al. 2015). Under high glucose conditions, SIRT3 binding to MPC1 is increased, resulting in reduced acetylation and increased pyruvate uptake (Liang et al. 2015). In hepatic cells, acetylation of MPC1 promotes its degradation, which limits pyruvate import and utilization in the liver (Vadvalkar et al. 2017). Finally, in type 1 diabetic Akita mouse hearts, increased acetylation of MPCs occurs without changes in protein abundance, which reduced pyruvate import and PDH activity (Li et al. 2018). Combined, these studies suggest that reduced MPC acetylation promotes mitochondrial pyruvate uptake, which would likely favor increased use of glucose‐derived metabolic products in oxidative respiration. As glucose‐driven mitochondrial respiration is decreased in GCN5L1‐depleted cells (which likely feature reduced global mitochondrial protein acetylation; Scott et al. 2012, 2014), this may argue for a mechanism other than aberrant mitochondrial pyruvate import as an explanation for the reduced bioenergetic output we observed (Fig. 4). Further work will be required to examine whether there is reduced glucose import at the cell membrane in GCN5L1‐depleted cardiac cells, or whether there is a compensatory upregulation of nonmitochondrial glycolytic pathways to aid in the metabolism of glucose in these cells.

In summary, we conclude that our findings are in line with the prevailing notion that under diabetic conditions, there is a move away from glucose oxidation in the diabetic heart, which is likely to involve a further reliance on fatty acids to provide ATP for contractile purposes (Fillmore et al. 2014). Future studies will likely focus on whether this switch is adaptive or maladaptive (Taegtmeyer et al. 2013; Fillmore et al. 2014), and whether novel therapeutics such as adropin (Gao et al. 2015; Thapa et al. 2019) can change the fuel substrate utilization patterns in the diabetic heart to improve function.

## Conflict of Interest

The authors declare that they have no competing interests associated with this manuscript.
